# lncRNA Gm16410 Mediates PM_2_._5_-Induced Macrophage Activation *via* PI3K/AKT Pathway

**DOI:** 10.3389/fcell.2021.618045

**Published:** 2021-03-16

**Authors:** Jingbin Xu, Henggui Xu, Kexin Ma, Yue Wang, Ben Niu, Li Zhang, Fasheng Li

**Affiliations:** ^1^Laboratory Medicine College, Dalian Medical University, Dalian, China; ^2^Department of Central Laboratory, Dalian Municipal Central Hospital Affiliated of Dalian Medical University, Dalian, China

**Keywords:** lncGm16410, PM 25, macrophage activation, inflammation, PI3K/Akt 3

## Abstract

PM_2_._5_ refers to atmospheric particulate matters with a diameter of less than 2.5 μm. The deposit of PM_2_._5_ in lung cells can cause oxidative stress, leading to changes in macrophage polarity, which can subsequently cause pulmonary inflammation. Long-chain non-coding RNA (lncRNA) is a class of transcripts that regulate biological processes through multiple mechanisms. However, the role of lncRNA in PM_2_._5_-induced lung inflammation has not been established. In this study, the biological effects and associated mechanism of lncRNA in PM_2_._5_-induced change in macrophage polarity were investigated. The lncRNA-mediated PM_2_._5_-induced macrophage inflammation and lung inflammation-associated injury were also determined. Mice were exposed to chronic levels of PM_2_._5_, and changes in the expression of lncRNA in the lung were measured by lncRNA microarray. lncRNAs that showed significant changes in expression in response to PM_2_._5_ were identified. lncRNA showing the biggest change was subjected to further analysis to determine its functional roles and mechanisms with respect to macrophage activation. The result showed that a significant reduction in expression of one lncRNA, identified as lncGm16410, was observed in the lung of mice and RAW264.7 cells following exposure to PM_2_._5_. lncGm16410 suppressed PM_2_._5_-induced macrophage activation *via* the SRC protein-mediated PI3K/AKT signaling pathway. PM_2_._5_ promoted lung inflammation by downregulating the expression of lncGm16410, enhancing the activation of macrophages. Thus, lncGm16410 might provide new insight into the prevention of PM_2_._5_ injury.

## Introduction

Pollution of the environment by fine particulate matter (PM_2_._5_, aerodynamic diameter ≤ 2.5 μm) has become one of the major issues to threaten public health (Gao et al., 2019). PM_2_._5_ contains mainly polycyclic aromatic hydrocarbons, transition metals, and endotoxin, all of which are known toxic chemicals (Deng et al., 2013; Ming et al., 2017). These particles can easily translocate from the alveoli to the bloodstream to affect the cardiovascular system, so long-term exposure to high concentrations of PM_2_._5_ increases the risk of cardiovascular and respiratory illnesses (Huang et al., 2017; Kirrane et al., 2019). In recent years, much progress has been made in the prevention and control of PM_2_._5_, but PM_2_._5_ remains a potential risk factor (Xie and Li, 2019). Despite the plethora of studies that have been conducted to investigate the toxicity of PM_2_._5_, the exact mechanism of PM_2_._5_-induced injury is still difficult to ascertain.

Long non-coding RNAs (lncRNAs) are a set of non-protein coding RNAs with a minimum length of 200 bases. lncRNAs modulate biological processes that are crucial for the growth and differentiation of cells as well as for the development of tumors (Ren et al., 2018). Recently, several studies have found that lncRNAs participate in the metastasis of tumor cells by competitively regulating miRNAs (Lin C. et al., 2018). For example, lncRNA AFAP1-AS1 was found to be the antisense DNA strand of the AFAP1 encoding gene locus, which acts as an adaptor protein that regulates actin integrity and links members of the SRC family proteins and other signaling proteins to actin filaments (Wu et al., 2013). These findings support the hypothesis that lncRNAs play an important role in various biological processes in cells injured by PM_2_._5_.

The main effects of PM_2_._5_ exposure on cardiopulmonary diseases are inflammatory response and oxidative stress, probably due to the stimulation of macrophages by PM_2_._5_ particles deposited in the alveoli and the release of some oxidants and cytokines. Several studies have demonstrated that the PI3K/AKT signaling pathway plays an important role in the activation of macrophage (Liu et al., 2017). Therefore, it has become important to investigate the role of lncRNAs in the process of cardiopulmonary disease caused by macrophage activation and the effect of lncRNAs on PM_2_._5_-regulated macrophage inflammatory response.

Most previous studies on PM_2_._5_ used tracheal instillation, an approach that is different from and that does not simulate the real situation of human exposure. In this study, a dynamic poisoning cabinet was used to construct a mice model, to determine changes in the expression of lncRNAs in mice in response to chronic PM_2_._5_ exposure and establish the role of lncRNA in PM_2_._5_-induced lung injury and inflammation. The findings suggested that lncRNA could reduce the extent of PM_2_._5_-induced lung inflammation and suppress PM_2_._5_-induced activation of macrophage.

## Materials and Methods

### Reagents

PrimeScript^TM^ RT reagent kit with gDNA Eraser (Perfect Real Time) (Code No.: RR047A)and SYBR^®^ Premix Ex Taq^TM^ II (Tli RNaseH Plus) (Code No.: RR820A) were purchased from TaKaRa. BCA protein assay kit (KGP902) and ECL detection kit were purchased from KeyGENE (Nanjing, China). Cell culture dishes were purchased from Guangzhou Jet Bio-Filtration Co., Ltd. (Guangzhou, China). Antibodies against β-actin (60008-1-lg, mouse), interleukin (IL)-6 (21865-1-AP, rabbit), tumor necrosis factor alpha (TNF-α; 60291-1-lg, mouse), protein kinase B (AKT; 10176-1-AP, rabbit), phosphoinositide 3-kinase (PI3K; 60225-1-lg, mouse), inducible nitric oxide synthase (iNOS; 18985-1-AP, rabbit), arginase-1 (ARG1; 16001-1-AP, rabbit), SRC (11097-1-AP, rabbit), and IL-10 (20850-1-AP, rabbit) were obtained from Proteintech (Wuhan, China). Antibodies against IL-1β (bs-20448R, rabbit), IL-12 (bs-1789R, rabbit), and P-AKT (bs-0876R, rabbit) were purchased from Bioss (Beijing, China). Antibody against P-PI3K (4228, rabbit) was purchased from CST. Dasatinib inhibitor was obtained from MCE (New Jersey, United States). lipofectamine^TM^ 3000 Reagent was purchased from Thermo Scientific^TM^ (United States). Mouse IL-6 ELISA kit was purchased from Boster (Wuhan, China).

### Extraction and Composition of PM_2_._5_

PM_2_._5_ was collected from a large-capacity air sampler of Langfang (Hebei, China) from October 2015 to March 2016. The collection of PM_2_._5_ was performed as described previously (Wang et al., 2017). Refer to Supplementary Material 1 for the component analysis of PM_2_._5_.

### Animal Experiments and Experiment Design

All animal experiments were approved by the Animal Experimental Committee of Dalian Medical University. Male Balb/c mice (6 weeks old) were obtained from Changsheng Biotechnology Co., Ltd. (Shenyang, China) (Approval No.: 2015-009). The mice were housed in a clean room (23 ± 1°C and humidity of 45–55%) provided with conventional food and water and maintained under a diurnal cycle of 12-h light and 12-h darkness. To construct the PM_2_._5_ exposure model, 16 mice were divided into two groups with eight animals per group. The first group was kept as healthy control; the environment of the control group and the PM_2_._5_ content in the environment were provided in Supplementary Material 2. The second group was exposed to PM_2_._5_ (PM_2_._5_ group). The PM_2_._5_ exposure was performed using a dynamic poisoning cabinet (HOPE-MED8050, Tianjin Hepu technology co. Ltd) that simulated the atmospheric environment. According to the standard concentration limit of environmental PM_2_._5_ and the ventilation of mice (The body weight in mice is about 30 g, the tidal volume is about 0.1–0.23 ml/kg, and the respiratory frequency is 136–230 times/min, the volume of poisoning warehouse is about 0.3 m^3^). The exposure concentration of 300 μg/m^3^ used in this experiment is about 225 μg/m^3^ of human living ambient air environment PM_2_._5_ concentration. The concentration of PM_2_._5_ in the flowing air was 300 μg/m^3^ for 4 months exposure, and the mice were exposed for 5 h/day for 5 days/week. At the end of the exposure period, blood was taken from the eyeball, and the lung tissue was removed and stored directly in liquid nitrogen for further experiments.

### Cell Culture and CCK8 Assay

RAW264.7 and THP-1 cells were cultured in DMEM (Gibco, CA, United States) or 1640 (Gibco) supplemented with 10% fetal bovine serum (FBS), 100 U/ml penicillin, and 100 μg/ml streptomycin in humidified air at 37°C with 5% CO_2_. First, RAW264.7 cells were resuspended, then diluted to 5 × 10^4^ cells/ml, and then 100 μl of the cell suspension was added to each well of a 96-well plate. These cells were either control cells or those that overexpressed lncGm16410, and they were treated with different concentrations of PM_2_._5_ for a specific time, followed by the addition of 10 μl of Cell Counting Kit-8 (CCK8) reagent and incubation at 37°C and in the dark for 2 h. After, the OD value of each well was measured by absorbance at 450 nm. Cell survival rates were expressed relative to that of the control.

### lncRNA Microarray Analysis

Microarray profiling was carried out by Shanghai Kangcheng Biotechnology Company. Briefly, the lung sample was labeled using Arraystar RNA Flash Labeling Kit, and a hybridization experiment was prepared using the Agilent SureHyb. After the chip was washed, it was scanned by an Agilent DNA Microarray Scanner, and the chip probe signals were acquired using the Agilent Feature Extraction software (version11.0.1.1). The Agilent GeneSpring GX v12.1 software was used to standardize the chip and to select lncRNAs that displayed differential expression.

### Plasmid Construction and Transfection

The plasmid that overexpresses lncGm16410 was designed and synthesized by Gene Pharma (Suzhou, China). Refer to Supplementary Material 3 for details.

### RNA Isolation and qPCR

The sequences of all primers used and details are listed in Supplementary Material 4.

### Western Blotting

Proteins were extracted from the lung tissue as previously described (Zhang et al., 2018a). Refer to Supplementary Material 5 for details.

### Enzyme-Linked Immunosorbent Assay (ELISA)

The expression of IL-6 in the serum was detected with an Enzyme-Linked Immunosorbent assay (ELISA) Kit (Boster, Wuhan, China). Blood samples were collected from the eyeballs of the animals and allowed to clot for 30 min at room temperature. The samples were then centrifuged at high speed, and the resulting serum from each sample was removed and stored it at −80°C.

### Flow Cytometry

Cells undergoing apoptosis were counted by flow cytometry using the annexin V-FITC apoptosis kit. The cells were first washed several times with PBS and then resuspended in binding buffer. Annexin V-FITC (5 μl) and propidium iodide (5 μl) were successively added to the cell suspension followed by incubation in the dark and at room temperature. Apoptotic rates were calculated as percentages of cells in the early plus late stages of apoptosis.

### Immunofluorescence and Immunohistochemistry

Refer to Supplementary Material 6 for details.

### Statistical Analyses

Data were reported as means ± standard error of the means (SEM). Statistical analysis was performed using GraphPad Prism version 7. Student’s *t*-test was used to compare data between two groups whereas a one-way analysis of variance was used to compare data from more than two groups. Statistical significance was considered at the *P* < 0.05 level.

## Results

### Differential Expression of lncRNAs in the Lungs of Mice Exposed to PM_2_._5_

**FIGURE 1:**
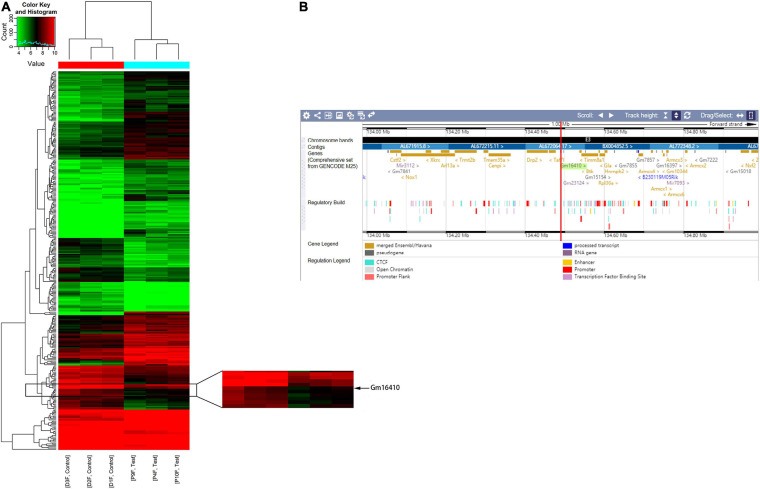
Changes in the expression of lncRNAs in the lung of mice following exposure to PM_2_._5_. **(A)** Hierarchical clustering analysis of lncRNAs that were differentially expressed in lung tissues of control and PM_2_._5_ exposed mice; each group contained three individuals. Red dots indicate above median expression level whereas green dots indicate below the median level across all samples (*n* = 3). **(B)** The genomic location of the lncGm16410 in the Ensembl database.

To determine whether lncRNAs were involved in the development of lung injury caused by exposure to PM_2_._5_, a DNA microarray was used to compare the expression profiles of lncRNA in the lungs of mice exposed to PM_2_._5_ with those in the lungs of unexposed mice. Of these lncRNAs that were expressed in the lung of the control group, a total of 201 lncRNAs were upregulated while 106 lncRNAs were downregulated in the lung of the PM_2_._5_ group (Figure 1A). Among these lncRNAs that showed different expression levels in the PM_2_._5_ group, lncGm16410 was significantly downregulated (Supplementary Figure 1), and the genomic location of the lncGm16410 was shown (Figure 1B). As our previous study has shown, PM_2_._5_ can induce macrophage exudation and lung tissue inflammation and injury (Wang et al., 2017). In the present study, the effect of lncGm16410 on these processes and the possible underlying mechanism was then determined.

### Expression of lncGm16410 Was Downregulated in PM_2_._5_-Exposed Macrophages

**FIGURE 2:**
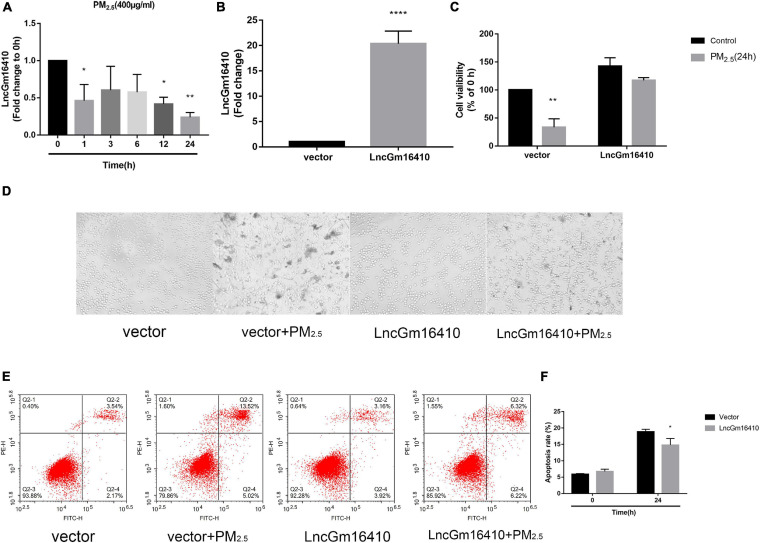
Effect of PM_2_._5_ on the expression of lncGm16410 in RAW264.7 cells. Control RAW264.7 cells and those overexpressing lncGm16410 were subjected to apoptosis assay by different methods. **(A)** Real-time quantitative PCR analysis of lncGm16410 transcript levels in the cells after being exposed to PM_2_._5_ for different times (*n* = 3). **(B)** Real-time quantitative PCR analysis of lncGm16410 that can optimize the transfection efficiency (*n* = 5). **(C)** CCK8 assays to determine the survival rate of macrophages exposed to PM_2_._5_ after they were transfected with lncGm16410 (*n* = 3). **(D)** Microscopic analysis of the growth status of macrophages exposed to PM_2_._5_. **(E**,**F)** Quantitative analysis of apoptosis (*n* = 3). Data are the means ± SEMs from at least three independent experiments, each performed in triplicate. *P < 0.5, **P < 0.01, and ****P < 0.0001—levels of significance that are different from the control group.

Based on accumulating evidence, alveolar macrophages are the effector cells of lung disease (Ma et al., 2017). Therefore, RAW264.7 macrophages were used to investigate the injury caused by PM_2_._5_ exposure, and the effect of PM_2_._5_ on lncGm16410 expression was measured in RAW264.7 macrophages. Consistent with the lncGm16410 expression level in the lung tissue, the expression of lncGm16410 in RAW264.7 cells decreased 24 h after exposure to PM_2_._5_ (Figure 2A). Simultaneously, the result showed that after 24 h of LPS treatment (5 μg/ml), the expression of lncGm16410 decreased significantly, which was similar to the results after PM_2__.5_ exposure (Supplementary Figure 2). Overexpression of lncGm16410 in RAW264.7 cells was used to access whether lncGm16410 expression correlated with PM_2_._5_-induced changes in macrophage activity. The level of lncGm16410 was significantly upregulated in RAW264.7 cells that overexpressed it (Figure 2B). In the control group, the survival rate of RAW264.7 cells was significantly reduced after exposure to PM_2_._5_, but RAW264.7 cells that overexpressed lncGm16410 appeared to exhibit a better survival rate following exposure to PM_2_._5_ (Figure 2C). Thus, overexpression of lncGm16410 appeared to protect the cells against apoptosis, thereby enhancing viability despite exposure to PM_2_._5_ for 24 h (Figures 2D–F). Taken together, these results indicated that lncGm16410 was involved in the regulation of cell growth when the macrophages were exposed to PM_2_._5_.

### lncGm16410 Regulates Macrophage Activation Under PM_2_._5_ Exposure

**FIGURE 3:**
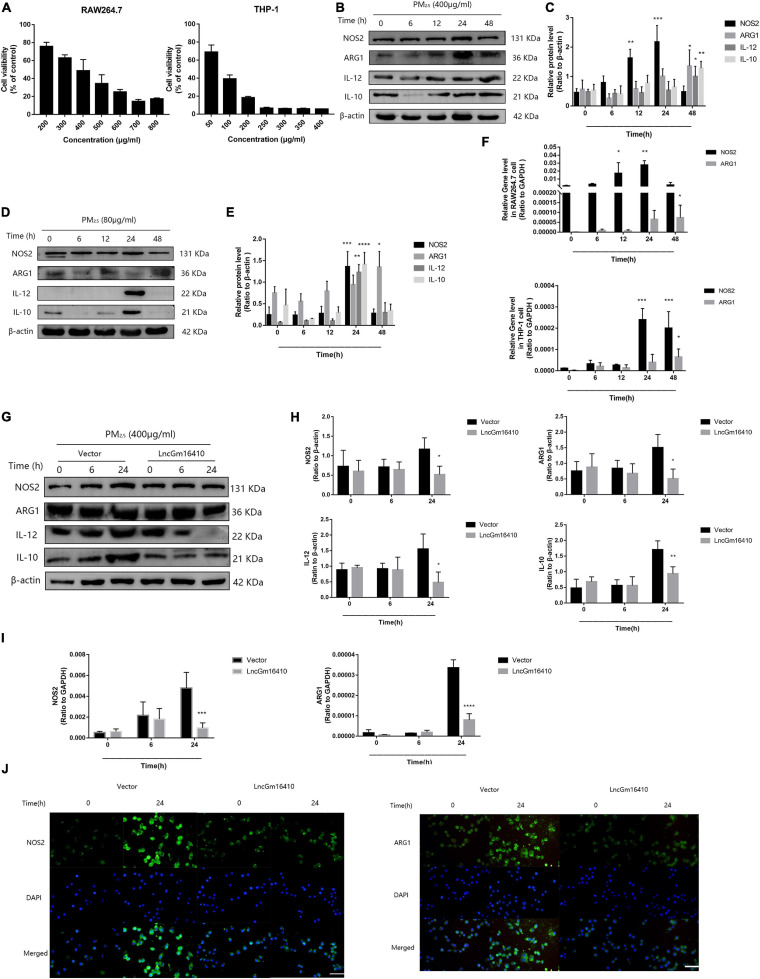
Effect of lncGm16410 overexpression on the activation of RAW264.7 cells following exposure to PM_2_._5_. **(A)** Survival rates of RAW264.7 (*n* = 6) and THP-1 (*n* = 4) cells after exposure to different concentrations of PM_2_._5_. **(B**,**C)** Western blot analysis of the M1 marker NOS2 and IL-12 and the M2 marker ARG1 and IL-10 in RAW264.7 cells. The plot gives a quantitative gray-scale analysis of the blot (*n* = 3). **(D**,**E)** Western blot analysis of the M1 marker NOS2 and IL-12 and the M2 marker ARG1 and IL-10 in THP-1 cells. The plot gives a quantitative gray-scale analysis of the blot (*n* = 3). **(F)** Real-time quantitative PCR analysis of NOS2 and ARG1 in RAW264.7 and THP-1 cells (*n* = 4). **(G**,**H)** Western blot analysis of the M1 marker NOS2 and IL-12 and the M2 marker ARG1 and IL-10 in RAW264.7 cells that overexpressed lncGm16410. The plot on the right gives a quantitative gray-scale analysis of the blot (*n* = 3). **(I)** Real-time quantitative PCR analysis of NOS2 and ARG1 in RAW264.7 cells after overexpression of lncGm16410 (*n* = 3). **(J)** Representative images of immunocytochemical staining analysis of the expression levels of NOS2 and ARG1 in RAW264.7 cells that overexpressed lncGm16410. Scale bar = 20 μm. All graphical data are the means ± SEMs from at least three independent experiments, each performed in triplicate. *P < 0.5, **P < 0.01, ***P < 0.001, and ****P < 0.0001—levels of significance that are different from the control group.

Recent studies have found that macrophages can be polarized into M1 or M2 subgroups upon activation by environmental stimulators, and the subsequent change in macrophage activity plays a significant role in the pathogenesis of pulmonary inflammation (Becerra-Diaz and Strickland, 2018; Li et al., 2018). First, the CCK8 method was used to determine the effect of PM_2_._5_ on the growth of RAW264.7 and THP-1 cells, and the median lethal dose was used to study the impact of PM_2_._5_ on macrophage activity. The median lethal doses of PM_2_._5_ for RAW264.7 and THP-1 cells were experimentally determined to be 400 and 80 μg/ml, respectively (Figure 3A). After 12 h of PM_2_._5_ exposure, the macrophage M1 marker protein was slightly upregulated, peaking after 24 h, while the macrophage M2 marker protein was upregulated after 48 h of exposure (Figures 3B,C). However, after 24 h of exposure, the macrophage M1 and M2 marker proteins in THP-1 macrophages were significantly upregulated (Figures 3D,E). Unsurprisingly, the results of qRT-PCR also demonstrated a significant increase in M1- and M2-related markers following PM_2_._5_ exposure (Figure 3F). Whether lncGm16410 might affect the PM_2_._5_-induced upregulated levels of M1 and M2 was then investigated. To test this, control RAW264.7 and those that overexpressed lncGm16410 were exposed to PM_2_._5_, and the changes in macrophage M1 and M2 levels were measured. After 24 h of exposure to PM_2_._5_, the mRNA and protein levels of M1 and M2 in the cells that overexpressed lncGm16410 were reduced (Figures 3G–I). Compared with the control cells, the increased expression of M1 and M2 proteins in the PM_2_._5_-treated cells was also detected by an immunofluorescence assay. Immunofluorescence assay also demonstrated that overexpression of lncGm16410 reduced the levels of M1 and M2 proteins in the cytoplasm (Figure 3J). These results indicated that lncGm16410 affected the phenotypic transformation of macrophages and confirmed that lncGm16410 could participate in the process of PM_2_._5_-induced macrophage activation.

### lncGm16410 Suppresses the Regulation of SRC Expression Under PM_2_._5_ Exposure

**FIGURE 4:**
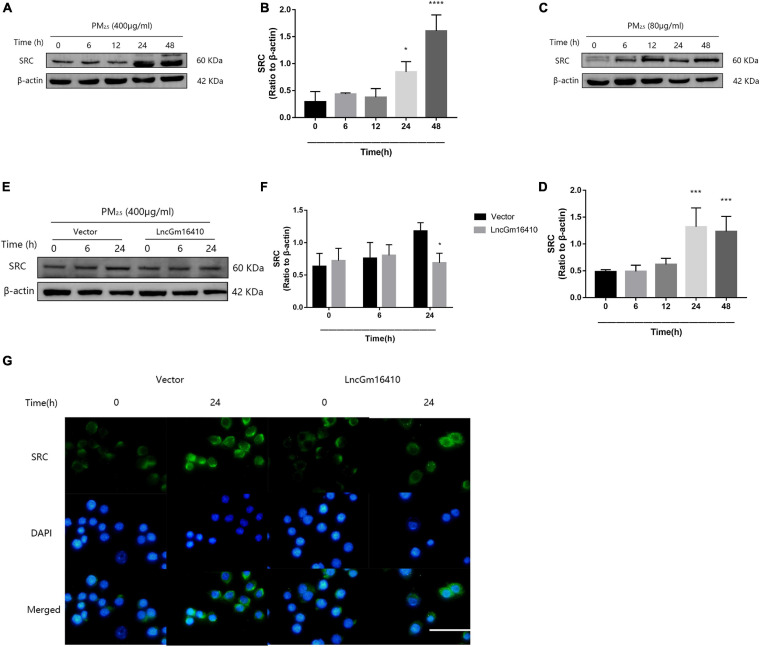
The expression of SRC was suppressed by lncGm16410 under PM_2_._5_ exposure. **(A**,**B)** Western blot analysis of SRC expression in RAW264.7 cells. The plot gives a quantitative gray-scale analysis of the blot (*n* = 3). **(C**,**D)** Western blot analysis of SRC expression in THP-1 cells. The plot gives a quantitative gray-scale analysis of the blot (*n* = 3). **(E**,**F)** Western blot analysis of SRC expression in RAW264.7 cells that overexpressed lncGm16410. The plot gives a quantitative gray-scale analysis of the blot (*n* = 3). **(G)** Representative images of the immunocytochemical staining analysis of SRC level in RAW264.7 cells that overexpressed lncGm16410. Scale bar = 20 μm. All graphical data are the means ± SEMs from at least three independent experiments, each performed in triplicate. *P < 0.5, ***P < 0.001, and ****P < 0.0001—levels of significance that are different from the control group.

According to the lncRNA microarray analysis, we found that, of the several genes adjacent to the lncGm16410, such as Taf7l, Timm8a1, and Btk, only Btk was slightly upregulated. The result was not statistically significant, which indicates that lncGm16410 may not act as a *cis*-acting gene. Recent studies have shown that exposure to particulate pollutants in the environment can lead to the activation of SRC protein-mediated signaling pathways, which in turn might trigger autophagy and inflammatory responses (Xu et al., 2016, Xu et al., 2019). To further investigate the changes in SRC protein expression in response to PM_2_._5_, the expression levels of SRC in RAW264.7 and THP-1 cells were measured following exposure to PM_2_._5_. Compared with the control cells, the levels of SRC protein in the PM_2_._5_-exposed cells displayed a rapid and sustained increased level of SRC after PM_2_._5_ treatment (Figures 4A–D), suggesting that PM_2_._5_ could indeed increase the expression of SRC. The effect of lncGm16410 on SRC expression in PM_2_._5_-exposed cells was then determined. The expression of SRC was attenuated in the cells that overexpressed lncGm16410 (Figures 4E,F). Changes in the expression of SRC were also confirmed by immunostaining in the cytoplasm of cells that overexpressed lncGm16410 (Figure 4G). lncGm16410 was involved in the regulation of PM_2_._5_-induced changes in SRC expression.

### SRC-Mediated Macrophage Activation *via* Activating PI3K/AKT Signaling

**FIGURE 5:**
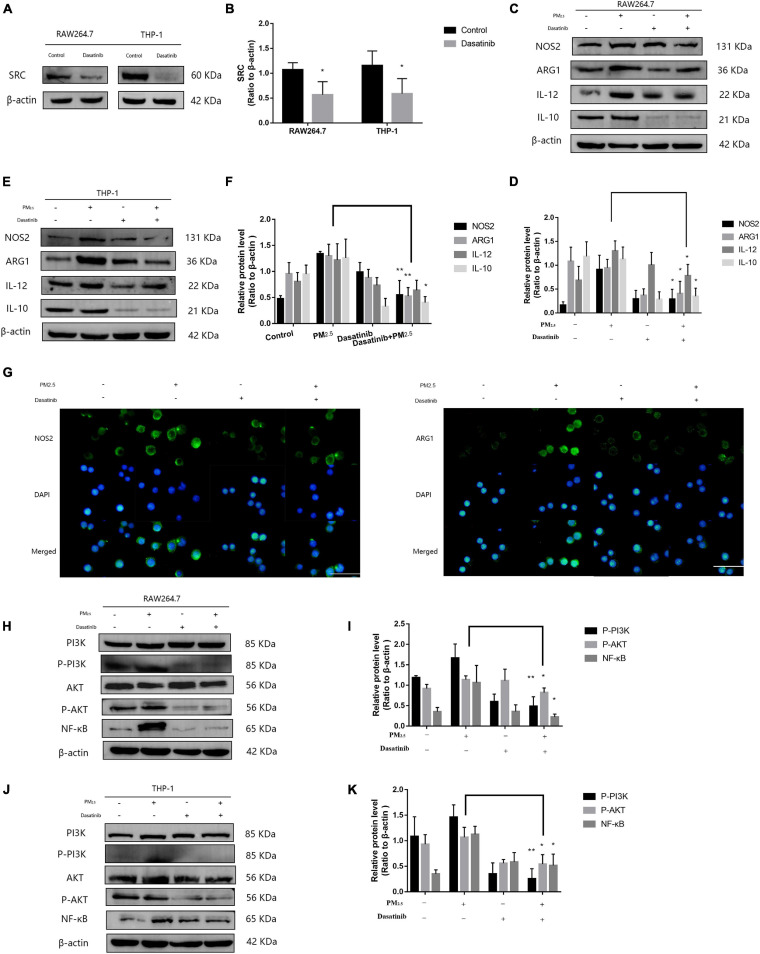
SRC-mediated macrophage activation *via* activating PI3K/AKT signaling. **(A**,**B)** Western blot analysis of SRC expression in RAW264.7 and THP-1 cells. The plot gives a quantitative gray-scale analysis of the blot (*n* = 4). **(C**,**D)** Western blot analysis of the M1 marker NOS2 and IL-12 and the M2 marker ARG1 and IL-10 in RAW264.7 cells. The plot gives a quantitative gray-scale analysis of the blot (*n* = 3). **(E**,**F)** Western blot analysis of the M1 marker NOS2 and IL-12 and the M2 marker ARG1 and IL-10 in THP-1 cells. The plot gives a quantitative gray-scale analysis of the blot (*n* = 3). **(G)** Representative images of immunocytochemical staining analysis of NOS2 and ARG1 levels in RAW264.7 cells. Scale bar = 20 μm. **(H**,**I)** Western blot analysis of P-PI3K, P-AKT, and NF-κB in RAW264.7 cells. The plot gives a quantitative gray-scale analysis of the blot (*n* = 3). **(J**,**K)** Western blot analysis of P-PI3K, P-AKT, and NF-κB levels in THP-1 cells. The plot gives a quantitative gray-scale analysis of the blot (*n* = 3). All graphical data are the means ± SEMs from at least three independent experiments, each performed in triplicate. *P < 0.5 and **P < 0.01—levels of significance that are different from the control group.

Although lncGm16410 suppressed SRC expression under PM_2_._5_ exposure, it is unclear whether SRC was involved in PM_2_._5_-mediated macrophage activation. Dasatinib is an inhibitor of SRC protein (Hermida-Prado et al., 2019). To determine whether SRC might be involved in PM_2_._5_-mediated macrophage activation, the cells were treated with dasatinib before they were exposed to PM_2_._5_ (Figures 5A,B), and the changes in macrophage polarity markers were measured. Compared with control cells, cells that were treated with dasatinib exhibited reduced levels of M1 and M2 expression as revealed by western blot (Figures 5C–F). The inhibitory effect of dasatinib on the expression of NOS2 and ARG1 was also confirmed by immunostaining (Figure 5G). The result indicated that SRC was involved in PM_2_._5_-induced macrophage activation. As an important molecule in the signal transduction pathway, SRC can shuttle between different cellular compartments to affect cellular processes by mediating the PI3K/AKT signaling pathway (Cheng et al., 2014; Cho et al., 2016). Therefore, to further study the mechanism by which SRC regulates macrophage activity upon exposure to PM_2_._5_, the expression levels of P-PI3K, P-AKT, and nuclear factor kappa B (NF-κB), proteins that are involved in the PI3K/AKT signaling pathway, were measured by western blot. Macrophages that were exposed to PM_2_._5_ displayed reduced expression of P-PI3K, P-AKT, and NF-κB when the cells were treated with dasatinib (Figures 5H–K), suggesting that SRC might be involved in the activation of the PI3K/AKT signaling pathway through regulating the phosphorylation of PI3K and AKT.

### lncGm16410 Alleviated Inflammation Caused by Macrophage Activation Under PM_2_._5_ Exposure

**FIGURE 6:**
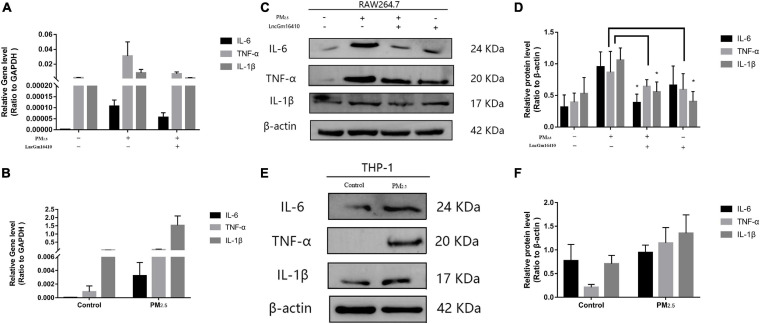
lncGm16410 regulate the inflammation response of macrophage induced by PM_2_._5_ exposure. **(A)** Real-time quantitative PCR analysis of IL-6, TNF-α, and IL-1β in RAW264.7 cells (*n* = 3). **(B)** Real-time quantitative PCR analysis of IL-6, TNF-α, and IL-1β expression in THP-1 cells (*n* = 3). **(C**,**D)** Western blot analysis of IL-6, TNF-α, and IL-1β transcripts in RAW264.7 cells. The plot gives a quantitative gray-scale analysis of the blot (*n* = 3). **(E**,**F)** Western blot analysis of IL-6, TNF-α, and IL-1β in THP-1 cells. The plot gives a quantitative gray-scale analysis of the blot (*n* = 3). All graphical data are the means ± SEMs from at least three independent experiments, each performed in triplicate. *P < 0.5 level of significance that is different from the control group.

According to accumulated evidence, the main consequence of macrophage activation is the secretion of related inflammatory factors (He et al., 2017; Zhang et al., 2018b). Therefore, changes in the levels of IL-6, TNF-α, and IL-1β of macrophages under PM_2_._5_ exposure were measured. Compared with the control cells, the expression of inflammatory factors in the PM_2_._5_-exposed cells was upregulated (Figures 6A–F). PM_2_._5_ exposure led to a slight decrease in the levels of related inflammatory cytokines in RAW264.7 cells that overexpressed lncGm16410 (Figure 6A). In addition, RAW264.7 cells that overexpressed lncGm16410 treated with PM_2_._5_ also displayed reduced levels of IL-6 and IL-1β (Figures 6C,D). These results indicated that overexpression of lncGm16410 may reduce inflammation by regulating macrophage activity upon exposure to PM_2_._5_.

### Macrophage Activation Caused Inflammation of Lung Tissue in Response to PM_2_._5_

**FIGURE 7:**
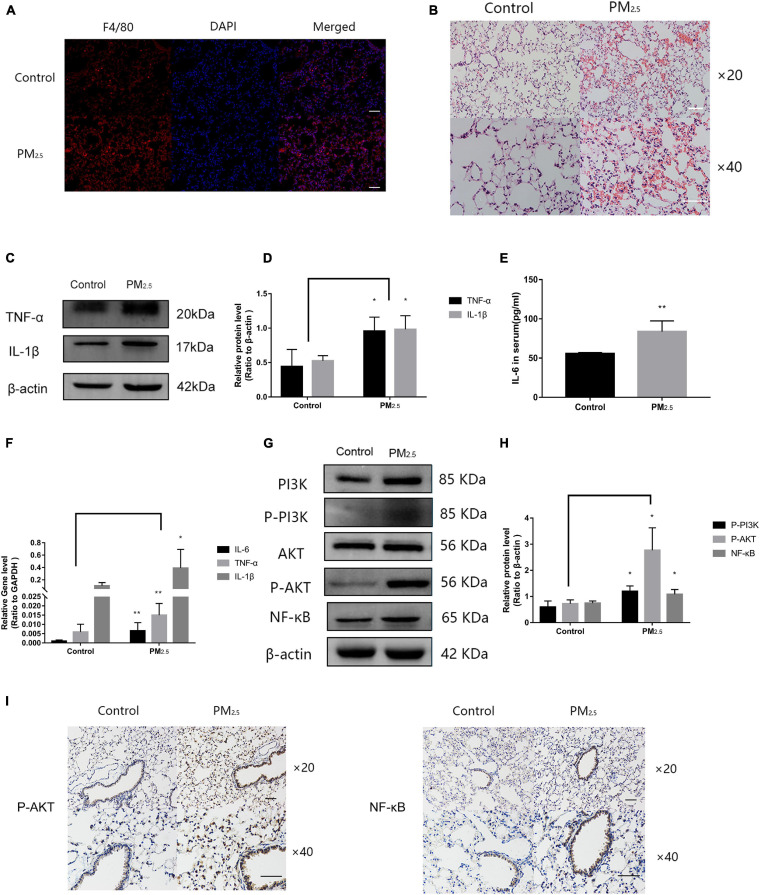
Macrophage activation caused inflammation of lung tissue in response to PM_2_._5_. **(A)** Immunohistochemistry detection of the macrophage marker F4/80 in mouse lung tissues. Scale bar = 10 μm. **(B)** Pathological changes in the lung tissues. Scale bar = 10 μm. **(C**,**D)** Western blot analysis of TNF-α and IL-1β levels in the lung tissues. The plot gives a quantitative gray-scale analysis of the blot (*n* = 3). **(E)** ELISA analysis of IL-6 in serum (*n* = 5). **(F)** Real-time quantitative PCR analysis of IL-6, TNF-α, and IL-1β in the lung (*n* = 8). **(G**,**H)** Western blot analysis of the P-PI3K, P-AKT, and NF-κB in mouse lung tissues. The plot gives a quantitative gray-scale analysis of the blot (*n* = 3). **(I)** Immunohistochemistry detection of P-AKT and NF-κB in mouse lung tissues. Scale bar = 10 μm. Data are the means ± SEMs from at least three independent experiments, each performed in triplicate. *P < 0.5 and **P < 0.01—levels of significance that are different from the control group at the levels, respectively.

Numerous studies have shown that PM_2_._5_ could induce lung tissue injury by activating macrophages (Feng et al., 2019). To determine the activation of macrophages in lung tissue under PM_2_._5_ exposure, immunofluorescence was applied to detect the macrophage marker F4/80 in the lung. Compared with the control mice, a large number of activated macrophages in the lung of mice exposed to PM_2_._5_ were observed (Figure 7A). In addition, for mice that were exposed to PM_2_._5_, histopathological examination of the lung tissue by H&E staining revealed a slight destruction of the alveolar wall and infiltration of inflammatory cells, including macrophages and lymphocytes, with red blood cells scattered in the alveolar areas (Figure 7B). To further characterize the inflammatory response *in vivo*, the levels of proteins and genes associated with inflammatory cytokines in the lung tissue were measured. Compared with the control group, the levels of TNF-α and IL-1β were significantly increased in response to PM_2_._5_ exposure (Figures 7C,D). Moreover, exposure to PM_2_._5_ also led to a significant increase of IL-6 levels in the serum (Figure 7E). The levels of TNF-α, IL-1β, and IL-6 were subsequently measured by *q*RT-PCR, which revealed obvious increases as a result of PM_2_._5_ exposure (Figure 7F). Finally, the link between the activation of the PI3K/AKT signaling pathway and PM_2_._5_-induced injury in the lung was investigated. The levels of PI3K, AKT, and NF-κB and their phosphorylation were upregulated in response to PM_2_._5_ exposure (Figures 7G,H). This finding was confirmed by immunohistochemical assay of P-AKT and NF-κB proteins. A large amount of brown-yellow fine particles was deposited in the section of the lung exposed to PM_2_._5_ (Figure 7I). Taken together, the results showed that macrophage activation in response to PM_2_._5_ exposure could cause lung tissue injury.

## Discussion

In recent years, PM_2_._5_, a major environmental pollution component, has attracted much attention because of its direct impact on health. Exposure to PM_2_._5_ can result in a variety of lung diseases and even brain injuries (Huang et al., 2019; Tian et al., 2019). In this study, we collected the PM_2_._5_ sample from the air in Hebei Province, the most polluted city in China, and analyzed its composition. The heavy metals and aromatic hydrocarbons present in PM_2_._5_ are considered to be the main components of PM_2_._5_ toxicity. Next, we used a dynamic poison cabinet to simulate the state of PM_2_._5_ in the environment and used Balb/c mice to construct a PM_2_._5_ exposure model *in vivo*.

According to related research, the microvesicles released *in vitro* from LPS-primed alveolar macrophages carry a large amount of TNF-α and a small amount of IL-1β/IL-6, and alveolar macrophages interact with lung epithelial cells through these microvesicles (Soni et al., 2016a; Allard et al., 2018). When a PM_2_._5_ suspension is instilled into the trachea of mice, the microvesicles released by macrophages can induce an increase in the number of BALF neutrophils as well as an increase in the expression of related inflammatory proteins (Soni et al., 2016b). Many studies have been carried out to find a way to prevent PM_2_._5_-induced injuries in macrophages. However, the mechanism and process of these injuries are still unclear.

lncRNAs are widely expressed as non-coding RNA, and increasing evidence suggests that lncRNAs play a major role in human physiological and pathophysiological processes (Chen et al., 2018). lncRNA microarray analysis of lung tissue samples taken from PM_2_._5_-treated Balb/c mice revealed 201 upregulated and 106 downregulated lncRNAs in response to PM_2_._5_ exposure (Figure 1A). The same profile of lncRNA expression was observed in RAW264.7 macrophages following exposure to PM_2_._5_ (Figure 2A). This indicated that lncGm16410 may be involved in PM_2_._5_-induced injuries, both in the lung and in the macrophage. Indeed, the data obtained from the macrophage CCK8 and apoptosis experiments *in vitro* (Figure 2C), as well as microscopic analysis of the lung tissue, suggested that lncGm16410 might be involved in the activation of macrophages in response to the PM_2_._5_-induced injuries sustained by the lung.

Notably, we observed that the downregulation of lncGm16410 expression in macrophages was consistent with lncGm16410 expression in the lung. Our data suggested that the initiation of inflammation induced by PM_2_._5_ recruited monocytes to the lungs where they then differentiated into macrophages, ultimately initiating an inflammatory cascade. According to our data, the recruitment of a large number of macrophages to the lung not only increased the expression of lncGm16410 in the lung, but the PM_2_._5_-activated alveolar macrophages also caused rapid and sustained inflammation as well as the production of cytokines and chemokines. These cytokines and chemokines released by the macrophages then diffused into the lung, causing tissue damage.

Under the stimulation of environmental factors, macrophages will carry out two different forms of inflammatory and anti-inflammatory functions (Saradna et al., 2018). M1 macrophages participate in the classic activation pathway and mainly produce immune-stimulating cytokines and other inflammatory response effectors (Sica et al., 2015). M2 macrophages are mainly involved in the upregulation of ARG1 (Nandakumar et al., 2017; Tardito et al., 2019). They have the immunomodulatory capacity, and they also play a major role in tissue remodeling, tumor progression, and mediating the regression of inflammation (Sica and Mantovani, 2012). In the present study, macrophage M1 phenotype-associated proteins NOS2 and IL-12 were found to be abundantly expressed after 24 h of exposure to PM_2_._5_, suggesting that PM_2_._5_ might stimulate the macrophage to produce an inflammatory response. PM_2_._5_ also induced a slow but late increase in the levels of macrophage M2 phenotype-associated proteins ARG1 and IL-10, demonstrating that macrophages were fully activated upon PM_2_._5_ exposure (Figure 3B) and that both M1 and M2 macrophages were involved in macrophage injury induced by PM_2_._5_. This study mainly focused on the role of lncGm16410 in macrophage activation and showed that lncGm16410 prevented the changes in M1/M2 phenotypes and a decrease in cell viability induced by PM_2_._5_, indicating a preventative effect of lncGm16410 on macrophage activation. Furthermore, lncGm16410 was found to be involved in macrophage-mediated inflammation responses, suggesting a key role for lncGm16410 in pneumonia (Figures 6A,C).

A recent study on lncRNAs outlines the important roles of these RNAs in the regulation of SRC expression (Zhang S. et al., 2018). In the current study, the expression of SRC in macrophages was upregulated in response to PM_2_._5_ exposure. However, overexpression of lncGm16410 counteracted this effect (Figure 4E). One possible explanation for this could be that lncGm16410 downregulated the expression of SRC in macrophages. After exposure to PM_2_._5_, inhibition of SRC in macrophages resulted in different degrees of reduction in the expression of macrophage phenotypic proteins (Figures 5C,E). This suggested that SRC could regulate PM_2_._5_-induced macrophage activity. Taken together, these results verified that lncGm16410 affects macrophage activation by downregulating the expression of SRC in response to PM_2_._5_ exposure. SRC was recently found to be involved in PI3K/AKT signaling pathway (Lu et al., 2003; Riggins et al., 2003; Yang et al., 2017; Beadnell et al., 2018). Our data showed that activation of the PI3K/AKT signaling pathway was also inhibited when the activity of SRC was reduced by the inhibitor dasatinib (Figures 5H,J). Although further experiments are needed to clarify the detailed mechanism, the current result does suggest a connection between SRC and PI3K/AKT signaling pathway.

Based on a previous study from our laboratory, acute exposure to PM_2_._5_ can cause lung tissue inflammation and oxidative stress (Wang et al., 2017). However, mice exposed to PM_2_._5_ exhibited only a small amount of red blood cells and exudates in the alveoli in their lung tissue (Figure 7B). One possible explanation for this phenomenon could be that chronic exposure to PM_2_._5_ simulated in this experiment was not a traditional tracheal instillation method but was carried out in the form of dynamic exposure. Although this exposure method did not cause any obvious pathological changes in the lung tissue exposed to PM_2_._5_, it still resulted in some changes to the levels of inflammation-associated proteins and genes in the lung. According to the results of this study, we found that PM_2__.5_ exposure can induce the expression of inflammation-related factors in the murine- and human-derived macrophages (RAW264.7 cells and THP-1 cells) and mouse lung tissue, which may cause lung tissue injury and then lead to lung inflammation in mice. As this study adopted a dynamic PM_2__.5_ exposure method, mice were exposed to PM_2__.5_ through inhalation for 4 months, which can better simulate the long-term exposure of humans to PM_2__.5_ in the environment. Simultaneously, by comparing relevant epidemiological studies (Zhao et al., 2017; Lin H. et al., 2018; Qi et al., 2020) this study found that PM_2__.5_ exposure in the environment increases the risk of lung disease and affects the quality of life and life expectancy.

In *in vitro* studies, the macrophages were transfected with a plasmid that overexpressed lncGm16410. The results were consistent with the decrease in inflammatory cytokines observed in the lncGm16410-overexpressing cells (Figures 6A,C). The reduced mRNA and protein levels of inflammatory factors such as TNF-α shown by lncGm16410-overexpressing cells indicated that the lncGm16410 may suppress the level of inflammatory responses in the lung by reducing the expression of TNF-α in macrophages (Figure 7C). These results firmly demonstrated the involvement of lncGm16410 in macrophage activation and macrophage inflammation *via* PI3K/AKT.

**FIGURE 8:**
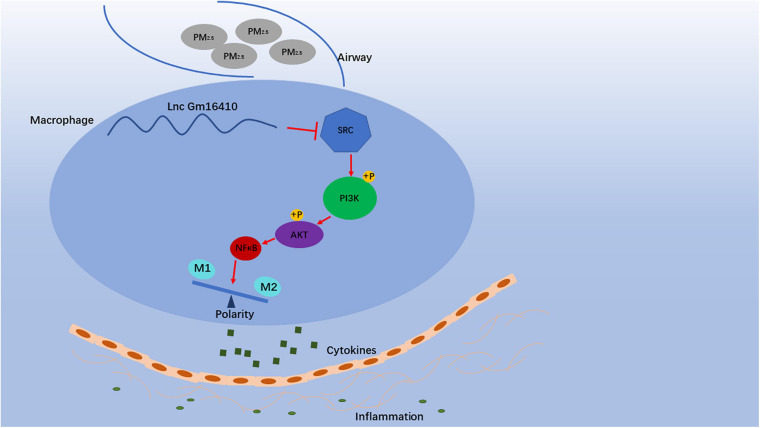
Schematic diagram showing the mechanisms through which lncGm16410 mediates PM_2_._5_-induced macrophages activation. When macrophage is exposed to PM_2_._5_, lncGm16410 suppresses the regulation of SRC expression, leading to a subsequent increase in the latter. The interactions among lncGm16410, SRC, and PI3K/AKT will dictate the balance of M1/M2 macrophages. Macrophage apoptosis and activation result in the overproduction of inflammatory factors and their subsequent release into the lung. This process, in turn, causes inflammation in the lung.

Our analyses of PM_2_._5_-exposed mice suggested that a decrease in lncGm16410 expression coincides with macrophage apoptosis and activation (Figure 8). Thus, both *in vivo* and *in vitro* results supported the speculation that lncGm16410 may serve as a potential marker of pneumonia caused by PM_2_._5_ and may be considered a new therapeutic strategy for lung inflammation.

## Data Availability Statement

The raw data supporting the conclusions of this article will be made available by the authors, without undue reservation.

## Ethics Statement

The animal study was reviewed and approved by all animal experiments were approved by the Animal Experimental Committee of Dalian Medical University.

## Author Contributions

JX and HX were responsible for the overall organizing of the experiments. JX, HX, KM, YW, and BN performed the experiments. JX wrote the manuscript. FL and LZ designed the experiments. All the authors read and approved the final manuscript.

## Conflict of Interest

The authors declare that the research was conducted in the absence of any commercial or financial relationships that could be construed as a potential conflict of interest.
