# Supplementary material for: lncRNA Gm16410 Mediates PM2.5-Induced Macrophage Activation via PI3K/AKT Pathway
Source: Front Cell Dev Biol. 2021 Mar 16;9:618045. doi: 10.3389/fcell.2021.618045 (PMC8007886; doi:10.3389/fcell.2021.618045)
Supplement: Supplementary file 1 [file Presentation_1.pdf]

Supplementary Material

LncRNA Gm16410 mediates $PM_{2.5}$-induced macrophage activation via PI3K/AKT pathway

Jingbin Xu[1, #], Henggui Xu[1, #], Kexin Ma[1],Yue Wang[1], Ben Niu[1], Li Zhang[2,*] , Fasheng Li [1,*]

[#]These authors contributed equally to this work

* Fasheng Li: E-mail: lifasheng@dmu.edu.cn

Li Zhang: E-mail: tyouri19652004@hotmail.com

Supplementary Material

1. The composition of $PM_{2.5}$

The components of $PM_{2.5}$ samples are shown in Table 1, mainly including organic carbon, elemental carbon, water-soluble ions and metal elements, etc.

Table 1. The composition and average concentration of $PM_{2.5}$ sample (ppm)

constituents		content	constituents	content	unit
organic carbon		1.1	elemental carbon	5.4	%
water-soluble ions	Na^+	38483.51	NH_4^+	3861.138	ppm
	K^+	4299.713	NO_3^-	35109.94	ppm
	Ca^{2+}	423.0402	SO_4^{2-}	41543.98	ppm
	Mg^{2+}	1933.556	Cl^-	16010.99	ppm
metal elements	Na	48347.69	Cu	103.11	ppm
	Mg	1404.46	Zn	3593.94	ppm
	Al	2493.27	As	23.05	ppm
	Ca	6245.23	Se	14.25	ppm
	V	8.44	pb	73.06	ppm
	Cr	51.18	Mo	41.47	ppm
	Mn	66.12	Cd	0.26	ppm
	Fe	1155.13	Ni	19.32	ppm
	Co	0.94			ppm

2. Animal experiments and experiment design

According to the principle of the dynamic poisoning cabinet, the control group received normal feeding, and the average content of $PM_{2.5}$ in the environment during the experiment was measured to be 21.3 μg/m^3, the components of $PM_{2.5}$ in the environment during the experiment are shown in Table 2, and organic matter has not been detected.

Table 2. The composition and average concentration of $PM_{2.5}$ (μg/m^3)

	constituents	content	constituents	content
	organic carbon	5.90	elemental carbon	1.92
water-soluble ions	Na^+	0.745	NH_4^+	7.27
	K^+	0.709	NO_3^-	5.00
	Ca^{2+}	0.368	SO_4^{2-}	7.47
	Cl^-	1.62		
metal elements	Na	0.364	Ni	0.0240
	Mg	0.980	Cu	0.137

	Ag	0.389	Zn	0.191
	Ca	0.226	As	0.0670
	V	0.0970	Se	0.0210
	Cr	0.169	Mo	0.987
	Hg	0	Pb	0.165
	Fe	0.377	Cd	0.675
	Co	0.0280	Sn	1.91

3. Plasmid construction and transfection

First, 70% confluence cells were collected by centrifugation and seeded in 6-well plates. When the cells reached 70% to 80% confluency, they were transfected with LipofectamineTM 3000 reagent. The lncGm16410-overexpressing plasmid and lipofectamine 3000 were mixed in serum-free medium, and the mixture was then added to the cells followed by incubation at 37 ℃ in 5% CO_2 for 24 h. After that, the medium was replaced with fresh DMEM supplemented with 10% FBS followed by additional incubation until the cells were almost 70% confluent. The transfected cells were selected using geneticin (G418). The medium was replaced with fresh DMEM containing 800 ng/μl of geneticin and 10% FBS, and the cells were cultured at 37 ℃ in 5% CO_2 for 24 hours. The cells were then washed twice with DMEM supplemented with 10% FBS.

4. RNA isolation and qPCR

Total RNA was extracted from the lung tissue and cells using TRIzol reagent (Invitrogen, Carlsbad, CA, USA). It was then used to synthesize the cDNA using a PrimeScript™ RT reagent kit with gDNA Eraser (Takara, Japan). Real-time PCR was performed with a TB GreenTM Premix Ex TaqTM Ⅱ (RR820A, Takara, Japan) and TP800 Thermal Cycle Dice (Applied Real-Time System). The relative expression levels of the tested genes were analyzed using the $2^{-\Delta Ct}$ and $2^{-\Delta\Delta Ct}$ method.

Table 3. Sequences of the primers used for qPCR

Gene	sequence
GAPDH (mouse)	F: AAATGGTGAAGGTCGGTGTGAAC<br>R: CAACAATCTCCACTTTGCCACTG
TNF-α (mouse)	F: GCCAGGAGGGAGAACAGAAACTC<br>R: GGCCAGTGAGTGAAAGGGACA
IL-1β (mouse)	F: TCCAGGATGAGGACATGAGCAC<br>R: GAACGTCACACACCAGCAGGTTA
IL-6 (mouse)	F: CCACTTCACAAGTCGGAGGCTTA<br>R: TGCAAGTGCATCATCGTTGTTC
NOS2 (mouse)	F: CAAGCTGAACTTGAGCGAGGA<br>R: TTTACTCAGTGCCAGAAGCTGGA
ARG1 (mouse)	F: AGCTCTGGGAATCTGCATGG

	R: ATGTACACGATGTCTTTGGCAGATA
GAPDH（homo）	F: GCACCGTCAAGGCTGAGAAC<br>R: TGGTGAAGACGCCAGTGGA
TNF-α (homo)	F: CACAGTGAAGTGCTGGCAAC<br>R: AGGAAGGCCTAAGGTCCACT
IL-1β (homo)	F: CAACAGGCTGCTCTGGGATT<br>R: CCATCATTTCACTGGCGAGC
IL-6 (homo)	F: GGCACTGGCAGAAAACAACC<br>R: GCTCTGGCTTGTTCCTCACT
NOS2（homo）	F: GCCAAGCTGAAATTGAATGAGGA<br>R: TTCTGTGCCGGCAGCTTTAAC
ARG1（homo）	F: CTGGCAAGGTGGCAGAAGTC<br>R: ATGGCCAGAGATGCTTCCAA

5. Western blotting

For the cultured cells, the plate containing the cells was washed three times with cold phosphate-buffered saline (PBS) followed by the addition of radioimmunoprecipitation assay (RIPA) buffer containing a mixture of protease inhibitors. The cells were incubated in this buffer for 10 min and the cell lysate was then transferred to a microcentrifuge tube. After centrifugation, the supernatant, which contained all the soluble proteins, was retained. Protein samples prepared from the lung tissue and cultured cells were subjected to SDS-PAGE using 10% or 12% gel. The proteins were transferred from the gel to a PVDF membrane, and the membrane was blocked with 5% non-fat dry milk for 2 h at room temperature followed by incubation with the designated primary antibodies overnight at 4 ℃. After that, the membrane was washed three times with TBS-Tween 20. Finally, the membrane was incubated with HRP-conjugated affinipure goat anti-mouse/rabbit IgG(H+L) secondary antibody (1:2000 dilution) for 2 h at room temperature, and the target protein bands on the membrane were detected by a chemiluminescence detection system. The intensity of each protein band was analyzed by ImageJ software.

6. Immunofluorescence and Immunohistochemistry

For cellular immunofluorescence, the cells were first spread on a glass slide and then fixed with 4% cold paraformaldehyde. The cells were then blocked with 5% BSA and incubated with a specific primary antibody at 4 ℃ overnight. After that, the cells were washed three times with PBS and incubated with the appropriate secondary antibody at room temperature for 30 min. Finally, the cells were stained with DAPI for 10 min, and slides were prepared for examination by fluorescence microscopy. For immunohistochemistry analysis, samples of the lung tissue were fixed with paraformaldehyde at 4℃ for 2 h and then washed with PBS, embedded and cut into 4-μm-thick sections on acid pre-treated slides. After dewaxing and repairing the antigen, the slides

were incubated with primary antibody at 4 ℃ overnight followed by incubation with HRP-labeled Goat Anti-Mouse IgG (H+L) at 37 ℃ for 30 min. The slides were finally observed with a fluorescence microscope.

Supplementary Figures

Figure1

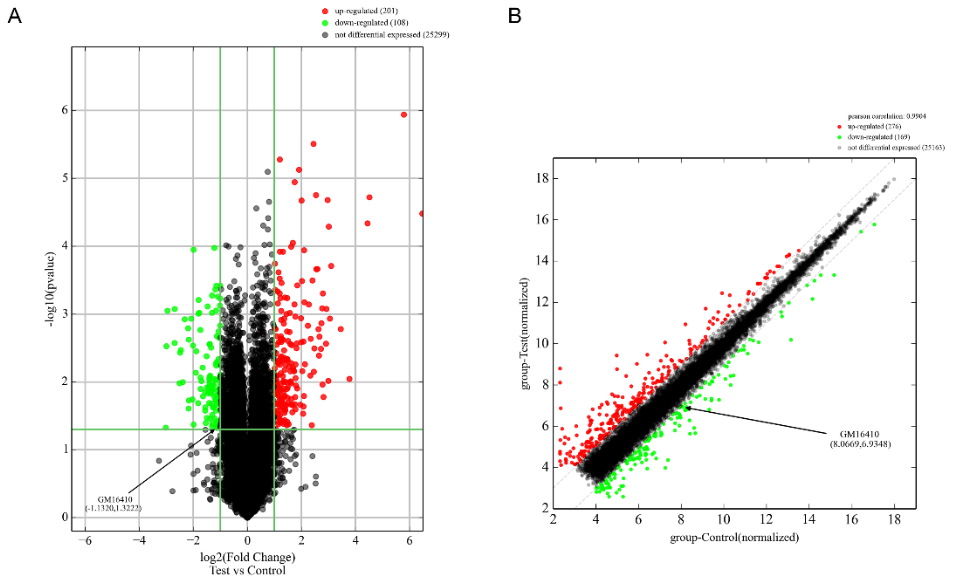

Figure 1. (A) Volcano plots were constructed using fold change values and P-values. The vertical line corresponds to a 2.0-fold up and down adjustment between the control and $PM_{2.5}$ groups, while the horizontal line represents the P value ≤ 0.05. The red and green dots in the figure represent the up-regulated and down-regulated lncRNA, respectively, and have statistical significance. (B) Scatter plots used to evaluate changes in lncRNA expression between the control and $PM_{2.5}$ treated samples. Both red and green dots show a greater than 2.0-fold changes between the control and $PM_{2.5}$ groups, where red points represent upregulation and green points represent downregulation. (n=3)

Figure2

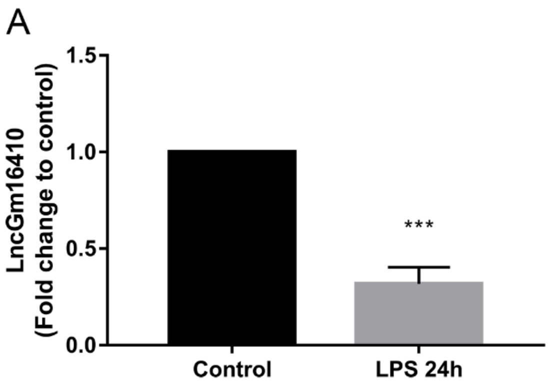

Figure 2. (A) Real-time quantitative PCR analysis of lncGm16410 transcript levels in the cells after being exposed to LPS (5μg/ml). (n=3)
